# Event and Comment

**Published:** 1921-10

**Authors:** 


### DENTAL REGISTER

THE MAGAZINE OF DENTISTRY

Vol. LXXV OCTOBER, 1921 No. 10

### EVENT AND COMMENT

**New School Prosthodontia.** For a few years there has been a new and appreciable interest developing in better prosthetic denture construction and adaptation. It probably first manifested itself when Dr. Green, by his strenuous propaganda, began to interest the profession in better impression taking with "Modeling Compound."

This pioneer work attracted the notice of Dr. Wm. A. Giffin, of Detroit, and he with the help of Mr. Kerr, of the Detroit Dental Manufacturing Co., they perfected a better modeling compound, and began a serious propaganda to make it practicable. Their perseverance has resulted in making this material and its use the standard procedure for denture construction at this time.

By the studies and works of Dr. Leon Williams, of New York, better porcelain teeth are now available; and, so the second advancement has resulted in a reason for making this a school of instruction and attainment.

The third step which has marked this development came when it was demonstrated that all seriously diseased and incurable teeth should be removed from the jaws. This requisition has precipitated the present demand for better plate denture service. This requirement has affected all classes of patients who are so unfortunate as to lose any considerable number of their natural teeth. Old and young alike have given up teeth that formerly would have been retained for service though considerably diseased. The exodontist has made great havoc with our precious dental organs, and we can not now glory in our gold inlays and crowns and bridges as we once did. We do not want to infer that all these former conceits and treasured glories have passed away; but at least some have received a severe shock, from which only time will demonstrate relief or a radical change in curative treatments.

At present we seem to have found consolation in attempts to provide intricate and useful methods of taking care of these losses made by extracting the teeth.

There has developed a so-called "New School of Prosthodontia," Which plans to construct and adopt more stable and serviceable plate dentures than we have ever made. It is hoped that our hopes may materialize, and that the science and art of our calling may be sufficient to cope with this terrible loss, and the great handicap which people generally will experience without their natural teeth.

We may be developing or going through some mysterious process of evolution which will at least be good for what seems to trouble us, but it doesn't seem logical. We have hoped for preventive dentistry, but it seems to be now a higher development of intricate technical and artistic prosthesis. So let it be, if no proper biologic and better technical interference can be discovered.

